# Serially transplantable mammary epithelial cells express the Thy-1 antigen

**DOI:** 10.1186/s13058-018-1006-y

**Published:** 2018-10-10

**Authors:** Neethan Amit Lobo, Maider Zabala, Dalong Qian, Michael F. Clarke

**Affiliations:** 1 Institute for Stem Cell Biology and Regenerative Medicine, School of Medicine, Stanford University, 265 Campus Drive, Stanford, CA 94305 USA; 2 Cell and Molecular Biology Program, University of Michigan, Ann Arbor, MI USA

**Keywords:** Tissue-specific stem cells, In vivo serial transplantation, Progenitor cells, Self-renewal

## Abstract

**Background:**

Recent studies in murine mammary tissue have identified functionally distinct cell populations that may be isolated by surface phenotype or lineage tracing. Previous groups have shown that CD24^med^CD49f^high^ cells enriched for long-lived mammary epithelial cells can be serially transplanted.

**Methods:**

Flow cytometry-based enrichment of distinct phenotypic populations was assessed for their gene expression profiles and functional proliferative attributes in vitro and in vivo.

**Results:**

Here, we show Thy-1 is differentially expressed in the CD24^med^CD49f^high^ population, which allowed us to discern two functionally different populations. The Thy-1^+^CD24^med^CD49f^high^ phenotype contained the majority of the serially transplantable epithelial cells. The Thy-1^−^CD24^med^CD49f^high^ phenotype contains a rare progenitor population that is able to form primary mammary outgrowths with significantly decreased serial in vivo transplantation potential*.*

**Conclusions:**

Therefore, Thy-1 expression in the immature cell compartment is a useful tool to study the functional heterogeneity that drives mammary gland development and has implications for disease etiology.

**Electronic supplementary material:**

The online version of this article (10.1186/s13058-018-1006-y) contains supplementary material, which is available to authorized users.

## Background

Mammary development is a highly ordered process that is regulated by spatiotemporal cues and directed by local and systemic signals [1]. Seminal studies in murine mammary cell biology revealed that the CD24^med^CD49f^high^CD29^high^ cell surface phenotype enriches for mammary repopulating units (MRUs) that have the greatest in vivo proliferative capacity, as measured by serial in vivo transplantation and differentiation into specialized cell types [2, 3]. However, the functional heterogeneity of phenotypically enriched pooled-cell populations complicates descriptions of such populations [4]. Therefore, there remains a need for better phenotypic markers to prospectively enrich for different functional cell populations that can be used to characterize these populations transcriptionally or precisely mark certain populations using lineage-tracing strategies. Thy-1, or CD90, is a GPI-anchored cell surface protein that was originally described as a mouse brain and thymus marker. Subsequent studies have shown that the Thy-1 antigen is expressed on many cell types, including hematopoietic stem cells (partially reviewed in [5, 6]). Thy-1 is expressed by basal cells in normal human mammary epithelium [7], where stem cells are thought to reside. Using the Thy-1^+^CD24^+^ phenotype, our group prospectively enriched for tumorigenic cells from a subset of MMTV-*Wnt1* murine mammary tumors that share properties with normal murine MRUs (mammary repopulating units, also known as stem cells) [8, 9]. Therefore, we sought to improve upon the current murine MRU cell surface phenotype by functionally assessing the prospective enrichment of serially transplantable mammary cells using Thy-1 expression. Our data revealed that Thy-1 expression on immature cells enriches for serially transplantable MRUs. Interestingly, the immature cells that lack Thy-1 expression enriched for a previously unknown rare population, which we term short-term mammary repopulating units (ST-MRUs), with limited serial proliferative potential in vivo.

## Methods

### Mouse strains

C57BL/6 and FVB mice were purchased from The Jackson Laboratory, Bar Harbor, ME, USA. pCx-GFP founder mice were kindly provided by Dr. Irving Weissman. All animals were maintained at the Stanford Animal Facility in accordance with the guidelines of both Institutional Animal Care Use Committees.

### Mammary gland dissociation and FACS

Six- to 10-week-old mice were euthanized and all fat pads surgically resected. Tissue was digested in Media 199 + 10 mM HEPES + PSA or L-15 for 2 h, and single-cell suspension was obtained as described previously [2] and then processed as described by the manufacturer’s instructions for Epicult (Stemcell Technologies, Vancouver, BC, Canada). For all experiments, cells were > 99% viable as assessed by Trypan Blue dye exclusion. Cells were then resuspended at a concentration of 1 × 10^7^ per ml and subjected to staining for flow cytometry. The antibodies CD24-PE, Thy-1.1-APC, Thy-1.1-PE-CY7, Thy-1.2-APC, and Thy-1.2-PE-CY7 were obtained from eBioscience (San Diego, CA, USA), CD45-BIO, Ter119-BIO, CD31-BIO, and CD140α-BIO were obtained from BD Pharmingen (San Jose, CA, USA), and Streptavidin-Pacific Blue was obtained from Invitrogen (Carlsbad, CA, USA). FACS for all experiments was performed on BD FACSAria II equipped with a UV laser. All gating to distinguish positive and negative expression was based upon IgG-isotype color-specific control staining.

### In vitro colony-forming assay

Ultra-low attachment 96-well plates (BD, Franklin Lakes, NJ, USA) were prepared with a feeder layer of irradiated L1-WNT3a mixed with 60 μl of growth factor-reduced Matrigel (BD) per well. Sorted cells were then plated into liquid media as previously described [10, 11] 10% FBS, 250 ng/ml Rspo I (R&D Systems, Minneapolis, MN, USA) and 2.5% growth factor-reduced Matrigel were added as supplements. 5 ng/ml purified mouse TGFβ1 ligand (R&D Systems) was added to wells as indicated. Colonies were counted after 7 days in culture in a 5% CO_2_ incubator.

### In vivo transplants

Sorted cell populations were collected in HBSS + 2% HICS and resuspended at the correct concentration before being injected into the cleared fat pads of 21–28-day-old recipient C57Bl/6 mice as previously described [2]. For all injections of 600 cells and below, cell counts were verified using either a nuclear staining count (1% Trypan Blue/0.1% Triton-X 100 in PBS) or GFP^+^ cell count. Cells were injected in either 10 or 5 ul volumes of PBS using a 25 ul Hamilton syringe. All transplants were allowed to grow for at least 5 weeks, but not more than 10 weeks before analysis. In the case of secondary and tertiary transplants, whole glands were dissected under fluorescence to obtain 1–2 mm pieces of tissue that contained GFP^+^ ductal structures that were transplanted into recipient mice.

### Whole mount and immunocytochemistry

Whole mounts stained with carmine alum were processed according to a standard protocol [12]. For immunocytochemistry, mammary glands were fixed in formalin and embedded in paraffin. Three-micrometer sections were dewaxed, hydrated and microwaved for 20 min in Tris-EDTA (0.01 M; pH 9) for antigen retrieval. Tissue sections were incubated overnight at 4 °C with primary antibodies in TBS + 1% BSA. Antibodies were CK6 (Covance, Princeton, NJ, USA), CK5 (Covance), Troma-I (DSHB, Iowa City, IA, USA) and Troma-III (DSHB). Sections were then incubated with anti-rat–Alexa A488 antibody and anti-rabbit–A594 antibody (both from Invitrogen) for 30 min at room temperature. Secondary antibodies were applied at 1:200 dilution. Samples were stained with DAPI and mounted in ProLong before pictures were taken. All images were produced with a Leica (Wetzlar, Germany) microscope and Image Pro Software (Media Cybernetics, Rockville MD, USA).

### RNA isolation and RT-PCR

Sorted cell populations were collected in staining media directly and then centrifuged at 5000 rpm for 5 min at 4 °C. Supernatant was then carefully removed from the cell pellet, which was immediately frozen in liquid N_2_ and stored at −80 °C until RNA extraction. RNA was extracted from frozen cell pellets by the Trizol method or MirVana kit (Life Technologies, Carlsbad, CA, USA). For RT-PCR, RNA was then converted to cDNA using the Superscript III Reverse Transcriptase system (Invitrogen). qPCR was then performed on fresh cDNA with 2 × SYBR Green Master Mix (Applied Biosystems, Foster City, CA, USA) according to the manufacturer’s instructions with primers to amplify specific genes (available upon request).

### Microarrays and expression analysis

RNA was then processed for microarray hybridization by the Stanford Protein and Nucleic Acids core facility. RNA was applied to Affymetrix (Santa Clara, CA, USA) GeneChip Mouse Genome 430 2.0 oligonucleotide arrays. The resulting CEL images were then processed using the TIGR TM4 [13, 14] suite of bioinformatic software. Arrays were pre-processed by MIDAS using robust multichip average (RMA) normalization across all samples. Using Cluster 3.0 [15], probes that expressed less than 6.229 (in Log_2_ space) in any two of the arrays and whose value was not at least fourfold different in any two arrays were excluded from the analyses. Probes intensities were then mean centered across all arrays as a set. Using MeV (TIGR), Rank products algorithm was then used to compare different populations using two-class unpaired comparisons with a critical *p* value of 0.01 for differentially expressed probes. Heatmaps are scaled from −3.0 (low) to 3.0 (high) in Log_2_ space. Microarray data may be found at GenBank under accession GSE89720.

## Results

### Thy-1 enriches for a molecularly distinct subset of CD24^med^CD49f^high^ cells

**Fig. 1:**
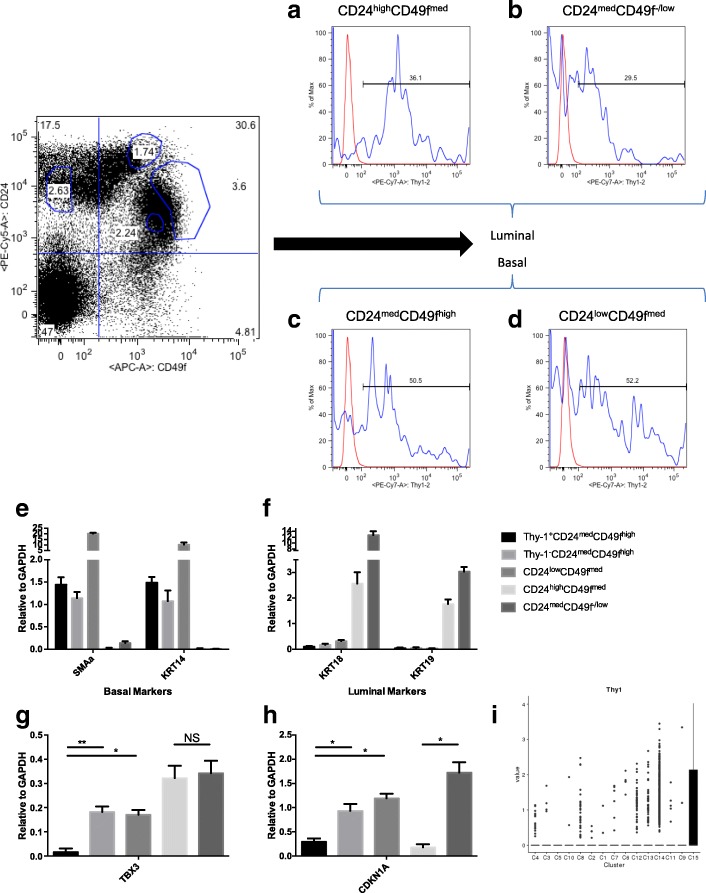
Thy-1 is differentially expressed in murine mammary epithelium. Freshly dissociated mammary epithelium analyzed for the expression of CD24 and CD49f. Thy-1 expression in (**a**) CD24^high^CD49f^med^ MaCFC luminal progenitor (**b**) CD24^med^CD49f^−/low^ luminal (**c**) CD24^med^CD49f^high^ and (**d**) CD24^low^CD49f^med^ myoepithelial cells. **e** Real-time PCR of sorted populations for basal marker genes’ expression. *N* = 3, ± STD. **f** Real-time PCR of sorted populations for luminal marker genes’ expression. N = 3, ± STD. **g** Real-time PCR of sorted populations for Tbx3 expression. N = 3, ± STD. **p < 0.01, *p < 0.05 for an unpaired two-tailed *t* test. **h** Real-time PCR of sorted populations for Cdkn1a (p21) expression. N = 3, ± STD. *p < 0.05 for an unpaired two-tailed *t* test. **i** Thy-1 expression in mouse mammary epithelial from single-cell RNA-seq data using the web tool (http://marionilab.cruk.cam.ac.uk/mammaryGland/) from Bach et al. [24]. C15 cells are annotated as Procr-enriched cells

Previous studies have shown CD49f and CD24 are cell surface markers that are able to distinguish self-renewing MRUs (CD24^med^CD49f^high^) from basal myoepithelial cells (CD24^low^CD49f^med^), CD24^high^CD49f^med^ colony-forming cells, and CD24^med^CD49f^−/low^ luminal cells [2, 3]. We found that Thy-1 was expressed on ~30% of luminal cell populations and ~50% of CD24^med^CD49f^high^ and myoepithelial cells (Fig. 1a, b, c, d). We also found that Thy-1 was expressed by CD24^med/high^CD133^+^Sca-1^+^ hormone-sensing luminal progenitor cells [16] as well as CD61^+^ luminal progenitor cells [17] (Additional file 1: Figure S1A). Real-time PCR confirmed the basal or luminal identity of sorted populations based on the expression of *Smaα* and *Krt14* (basal marker) and *Krt18* and *Krt19* (luminal marker) genes, respectively (Fig. 1e and f). To test if our enrichment procedures dramatically altered the physiology of the sorted populations, we exposed sorted populations to TGFβ1, a well-studied ligand that is associated with epithelial-to-mesenchymal transition [18]. Sorted populations that expressed basal or luminal gene markers shared similar responses to TGFβ1 exposure (Additional file 1: Figure S1B). Therefore, the sorting paradigm and related procedures effectively enriched for physiologically distinct phenotypic populations.

Numerous studies have linked the transcriptional expression of key genes to the function of cells enriched for by cell surface demarcation or lineage-tracing strategies [16, 19]. For example, Tbx3 is a gene that regulates the development of mammary hormone-sensing cells [20]. Interestingly, we found that all populations except for the Thy-1 expressing CD24^med^CD49f^high^ cells also expressed Tbx3 (Fig. 1g). This population also had a low transcriptional level of Cdkn1a (Fig. 1h), whose expression is associated with halted cell cycle progression, comparable to luminal CD24^high^CD49f^med^ cells that have been previously characterized to be highly proliferative. Furthermore, single-cell RNA-Seq data from the Marioni and Khaled laboratories showed that Thy-1 is most highly expressed in cells that are enriched for Procr, a Wnt target gene that has been linked to multipotent mammary stem cells [21] (Fig. 1i). Taken together, these results suggested that Thy-1 does indeed segregate a functionally distinct subpopulation of CD24^med^CD49f^high^ cells.

**Fig. 2:**
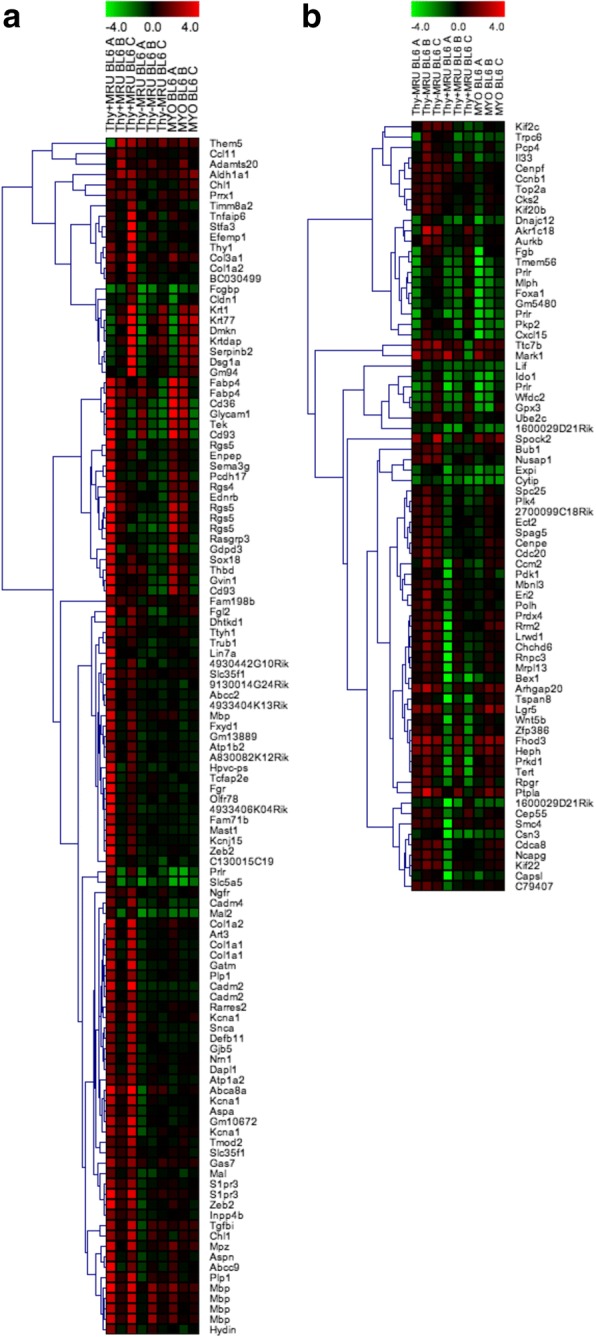
Microarray analysis of sorted mammary populations from virgin C57BL6 mice. Thy + MRU represents Thy-1^+^CD24^med^CD49f^high^, Thy-MRU represents Thy-1^−^CD24^med^CD49f^high^, and MYO represents CD24^low^CD49f^med^. **a** Differentially expressed probes comparing Thy-1^+^CD24^med^CD49f^high^ to both Thy-1^−^CD24^med^CD49f^high^ and CD24^low^CD49f^med^ basal populations. **b** Differentially expressed probes comparing Thy-1^−^CD24^med^CD49f^high^ to both Thy-1^+^CD24^med^CD49f^high^ and CD24^low^CD49f^med^ basal populations

To further investigate the transcriptional differences that define each population, we performed gene expression microarrays on sorted cell populations. We found that Thy-1^+^CD24^med^CD49f^high^, Thy-1^−^CD24^med^CD49f^high^, and CD24^low^CD49f^med^ cells had unique global gene expression profiles in both C57BL6 and FVB mice (Fig. 2a, b, Additional file 2: Figure S2A and B). There were both strain-specific and distinct genes expressed by sorted populations (Additional file 3). Notably, the Thy-1^+^CD24^med^CD49f^high^ cells expressed the highest level of genes that have previously been linked to mammary gland stem cells such as Rarres2 and Aldh1a1 [22, 23] (Fig. 2a). The enriched expression of Rarres1 and Aldh1a1 in both mouse strains’ microarray data in the Thy-1^+^CD24^med^CD49f^high^ cells was confirmed by real-time PCR and in Procr-enriched cells by single-cell RNA-seq from the Marioni and Khaled laboratories [24] (Additional file 1: Figure S1C, D, E, F). The Thy-1^+^CD24^med^CD49f^high^ population was also enriched for the expression of Procr by real-time PCR (Additional file 1: Figure S1E), with the Procr-enriched population expressing high levels of both Procr and Thy-1 by single-cell RNA-seq (Fig. 1i and Additional file 1: Figure S1H). We also observed that CD24^high^CD49f^med^ luminal cells expressed genes that distinguished this population from CD24^med^CD49f^−/low^ luminal cells in both the C57BL6 and FVB mouse strains (data not shown, Additional file 2: Figure S2C and Additional file 3). In the BL6 strain, the CD24^med^CD49f^−/low^ population was enriched for the expression of Elf5, a transcription factor that suppresses mammary stem cell activity [25] (Additional file 3).

### Thy-1 enriches for an extensively proliferating subset of CD24^med^CD49f^high^ cells

**Fig. 3:**
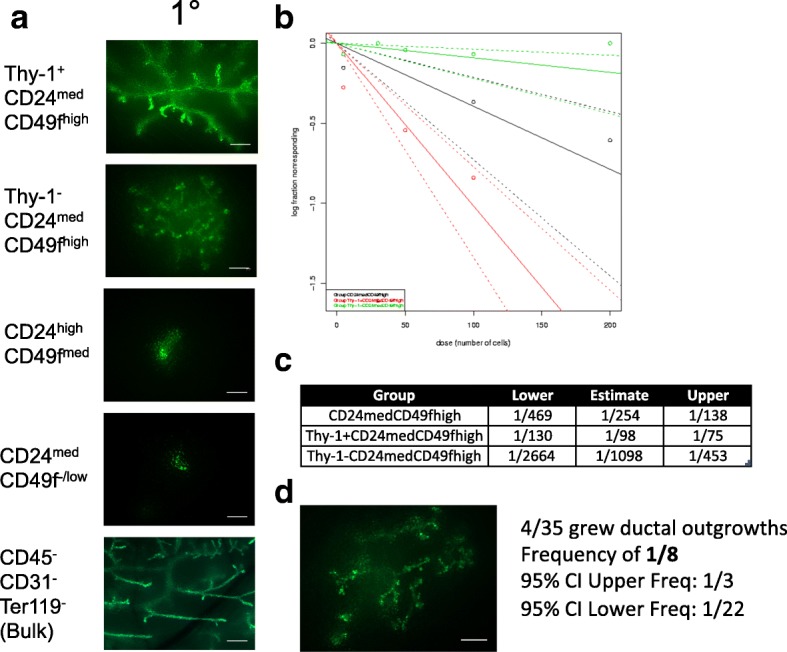
Thy-1^+^CD24^med^CD49f^high^ is enriched for ductal outgrowth-forming MRUs. **a** Representative images of primary transplanted ductal epithelium produced by 100 transplanted cells of the indicated GFP^+^ sorted population. Scale bars show 500 μm. **b** Summary of limiting dilution transplantation series. **c** Estimated frequency of ductal outgrowth-forming cells in the indicated transplanted population. **d** Representative image and frequency of ductal outgrowths from single-cell transplants of GFP^+^Thy-1^+^CD24^med^CD49f^high^. Scale bar shows 500 μm

Murine mammary stroma depleted of endogenous epithelium provides an excellent in vivo model system to assess the repopulating potential and frequency of implanted cell populations. We leveraged this system and transplanted sorted cell populations from pCx-GFP transgenic mouse cells that ubiquitously express GFP to distinguish donor outgrowths from any residual endogenous epithelium. We found that 100 Thy-1^+^CD24^med^CD49f^high^ and 100 Thy-1^−^CD24^med^CD49f^high^ cells were able to produce GFP^+^ ductal outgrowths that looked morphologically similar to those produced by 100 bulk mammary epithelial cells (MECs) (Fig. 3a). However, neither luminal CD24^high^CD49f^med^ progenitor cells, CD24^med^CD49f^−/low^ luminal cells, nor CD49f^med^CD24^low^ differentiated myoepithelial cells were able to produce extensive outgrowths up to 10,000 cells transplanted (Fig. 3a and data not shown). Limiting dilution transplantation studies revealed that the frequency of engrafting cells in the total CD24^med^CD49f^high^ population was 1 in 254 cells, consistent with previous reports (Fig. 3b, c, Additional file 4: Figure S3A). Strikingly, we observed that the majority of the outgrowth-forming ability was confined to CD24^med^CD49f^high^ cells that also expressed Thy-1 (Fig. 3b, c, Additional file 4: Figure S3A). Single-cell transplantation revealed that one in eight cells from the Thy-1^+^CD24^med^CD49f^high^ population were able to produce clonal outgrowths (Fig. 3d).

Previous reports using limiting dilution transplantation studies revealed that the MRU frequency within the CD24^med^CD49f^high^ phenotype might be influenced by the strain of mice employed [2, 3]. Therefore, we performed another set of limiting dilution transplantation experiments in wild-type (WT) FVB mice (Additional file 4: Figure S3B). Again, we found that CD24^med^CD49f^high^ cells that also expressed Thy-1 contained most of the outgrowth-forming cells (Additional file 4: Figure S3C). In FVB mice, the Thy-1^−^CD24^med^CD49f^high^ cells gave rise to smaller ductal outgrowths that were largely devoid of side branching structures (Additional file 4: Figure S3D). The frequency of outgrowth-forming cells in the Thy-1^+^CD24^med^CD49f^high^ population was 1 in 156 cells in FVB mice, similar to the frequency in C57BL6 mice (Additional file 4: Figure S3E).

### Thy-1^+^CD24^med^CD49f^high^ enriches for transplanted outgrowths with serial transplantation potential

**Fig. 4:**
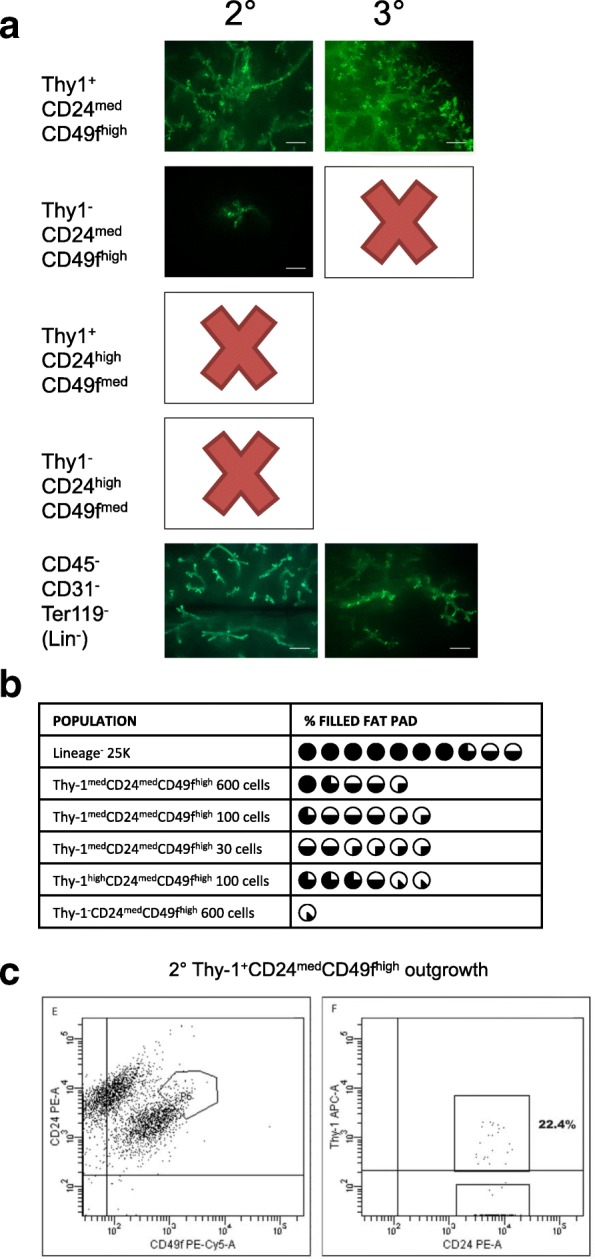
Thy-1^+^CD24^med^CD49f^high^ enriches for self-renewing MRUs. **a** Representative images of secondary and tertiary GFP^+^ ductal outgrowths produced from the indicated originally sorted populations. Scale bars show 500 μm. **b** Pie-chart representation of the secondary outgrowth formation of the indicated population as a percentage of the total surface area of the engrafted stromal mammary fat pad. **c** Flow cytometric analysis for CD24, CD49f, and Thy-1 expression of representative secondary GFP^+^ ductal outgrowth produced from a fragment of primary ductal outgrowth originated from GFP^+^Thy-1^+^CD24^med^CD49f^high^ engrafted cells

MRUs are defined as cells that have both the ability to self-renew and to differentiate into mature luminal and basal cells that give rise to functional milk-secreting epithelium. The presence of MRUs can be detected by serially passaging transplanted cells or tissue fragments in vivo since only cells with extensive proliferative capacity will be able to continually produce outgrowths [3, 26]. Our initial transplantation data showed that the Thy-1^+^CD24^med^CD49f^high^ phenotype contained most of the outgrowth-forming cells. We serially transplanted 2 mm^3^ fragments of GFP^+^ donor epithelium from the initial primary outgrowths that were produced by C57BL6 Thy-1^+^CD24^med^CD49f^high^, or Thy-1^−^CD24^med^CD49f^high^ GFP^+^ cells and found these outgrowths grew secondary GFP^+^ structures (Fig. 4a). A total of 23 out of 33 Thy-1^+^CD24^med^CD49f^high^ primary GFP^+^ epithelial fragments engrafted upon secondary transplantation, giving an engraftment efficiency of about 67% (Additional file 5: Figure S4A). We found that Thy-1^+^CD24^med^CD49f^high^ cells were able to give rise to secondary ductal outgrowths that resembled the outgrowths formed from bulk MECs and the phenotypic CD24, CD49f and Thy-1 diversity of WT glands (Figs. 1 and 4c). Thy-1^+^CD24^med^CD49f^high^ primary outgrowths were also able to give rise to tertiary outgrowths similar to bulk MECs (Additional file 5: Figure S4B). Strikingly, we found that only 11% of the GFP^+^ fragments produced by Thy-1^−^CD24^med^CD49f^high^ transplanted cells were able to form secondary outgrowths (Additional file 5: Figure S4A). In addition, the secondary outgrowths were much smaller than those produced by initially engrafted GFP^+^ Thy-1^+^CD24^med^CD49f^hi^ cells (Fig. 4a). Unlike the Thy-1^+^CD24^med^CD49f^high^ epithelium that produced tertiary outgrowths like bulk MECs, the few GFP^+^ secondary outgrowths formed by the initially engrafted GFP^+^Thy-1^−^CD24^med^CD49f^hi^ cells were unable to re-engraft a third time (Fig. 4a). These data demonstrated that Thy-1^+^CD24^med^CD49f^high^ phenotype enriched for MRUs and that the Thy-1^−^CD24^med^CD49f^hi^ phenotype cells were enriched for basal short-term MRUs (ST-MRUs) with less proliferative capacity.

Functionally competent mammary epithelium will be able to give rise to alveolar milk-secreting cells upon pregnancy. We took mice that were engrafted with secondary GFP^+^ outgrowths produced by bulk MECs and Thy-1^+^CD24^med^CD49f^high^ cells at 10 weeks posttransplantation and mated them to determine the milk-producing capability outgrowths. Hematoxylin and eosin staining showed extensive expansion of the ductal epithelium (Additional file 5: Figure S4C). Cytokeratin staining showed CK14, CK8, CK19, and CK6 localization was indistinguishable between WT (non-transplanted), primary, and secondary GFP^+^ transplanted epithelium produced by Thy-1^+^CD24^med^CD49f^high^ cells (Additional file 5: Figure S4C). In addition, Thy-1^+^CD24^med^CD49f^high^ secondary outgrowths underwent pregnancy morphogenesis in a similar fashion to WT epithelium as evaluated by cytokeratin expression, with visual confirmation of milk inside of the lumen of ducts in hematoxylin and eosin staining (Fig. 4c).

## Discussion

Prospective isolation studies have shown that mammary tissue contains a distinct cell population of MRUs that is enriched for the ability to establish a functionally competent mammary gland in serial transplantation assays [2, 3]. We found that the Thy-1^+^CD24^med^CD49f^high^ phenotype enriches for mammary cells with the highest proliferative capacity as measured by this assay. Surprisingly, we found single-cell transplantation revealed a highly enriched MRU frequency in the Thy-1^+^CD24^med^CD49f^high^ cells compared to pooled-cell transplants, which may be due to multiple progenitor cells competing for sparse niche components. We also noted that the Thy-1^+^CD24^med^CD49f^high^ cells expressed genes associated with these cells with extensive proliferation capacity [22]. Since the Thy-1^−^CD24^med^CD49f^high^ population contains cells with diminished capacity to serially form extensive ductal outgrowths in vivo, these cells may be comparable to multipotent progenitor cells in the hematopoietic system, which also have a lesser proliferative capacity compared to stem cells. To our knowledge, this is the first report of the existence of a mammary epithelial cell population with short-term MRU capacity. In addition to MRUs, unipotent progenitor cells have been identified and bipotent progenitor cells that lack in vivo transplantation capacity have been previously described [2, 27].

We found that the ortholog of TBX3, a gene mutated in ulnar mammary syndrome (UMS), was expressed in all populations except for Thy-1^+^CD24^med^CD49f^high^. Recent studies have shown that Tbx3 is required for the mammary development and generation of hormone-sensing cells in murine mammary epithelium [28, 29]. Our data suggests that the UMS breast phenotype may be a defect of aberrant specification of cells with the capacity to undergo terminal differentiation. Recent studies have also demonstrated that Elf5 expression is necessary for the differentiation of luminal progenitor cells [25]. Our data showing that the CD24^med^CD49f^−/low^ compartment expresses Elf5 suggests that this compartment participates in a feedback pathway that instructs mammary epithelial cells to proliferate and differentiate.

## Conclusions

Taken together, our data shows that the inclusion of Thy-1 expression to the growing list of phenotypic markers will allow further dissection of the mammary cellular hierarchy and allow for more accurate interpretation of pooled or single-cell analyses.

## Additional files

Additional file 1: **Figure S1.** Phenotypic and physiological analysis of sorted populations. (A) Flow cytometric analysis on luminal cells for Thy-1, CD133, Sca-1, and CD61 expression. (B) Exposure of cultured FACS-enriched mammary populations to TGFB1 ligand. The indicated sorted mammary populations were cultured in three-dimensional self-renewing conditions with and without the addition of 5 ng/ml human TGFB1 ligand. *N* = 3, ± STD. (C) Real-time PCR of sorted populations for Procr expression. N = 3, ± STD. *-*p* < 0.05 for an unpaired two-tailed *t* test. (D) Procr expression in mouse mammary epithelial from single-cell RNA-seq data using the web tool (http://marionilab.cruk.cam.ac.uk/mammaryGland/) from Bach et al. [24]. C15 cells are annotated as Procr-enriched cells. (E) Real-time PCR of sorted populations for Rarres2 expression. N = 3, ± STD. *p < 0.05 for an unpaired two-tailed *t* test. (F) Rarres2 expression in mouse mammary epithelial from single-cell RNA-seq data using the web tool (http://marionilab.cruk.cam.ac.uk/mammaryGland/) from Bach et al. [24]. C15 cells are annotated as Procr-enriched cells. (G) Real-time PCR of sorted populations for Aldh1a1 expression. N = 3, ± STD. *p < 0.05 for an unpaired two-tailed *t* test. (H) Aldh1a1 expression in mouse mammary epithelial from single-cell RNA-seq data using the web tool (http://marionilab.cruk.cam.ac.uk/mammaryGland/) from Bach et al. [24]. C15 cells are annotated as Procr-enriched cells. (PDF 293 kb)

Additional file 2: **Figure S2.** Microarray analysis of phenotypically enriched populations isolated from virgin FVB female mice. (A) Differentially expressed probes comparing Thy-1^+^CD24^med^CD49f^high^ to both Thy-1^−^CD24^med^CD49f^high^ and CD24^low^CD49f^med^ basal populations. (B) Differentially expressed probes comparing Thy-1^−^CD24^med^CD49f^high^ to both Thy-1^+^CD24^med^CD49f^high^ and CD24^low^CD49f^med^ basal populations. (C) Differentially expressed probes comparing CD24^high^CD49f^med^ to CD24^med^CD49f^−/low^ luminal cells. (PDF 186 kb)

Additional file 3: Strain and sorted population-specific and overlapping gene sets. Using the microarray data from C57BL6 and FVB mouse strains, each population’s significantly enriched genes’ expression was compared to determine the overlapping genes as well as the strain-specific genes. File contains tabular sheets of data that correspond to Thy-1^+^CD24^med^CD49f^high^, Thy-1^−^CD24^med^CD49f^high^, CD24^low^CD49f^med^, CD24^high^CD49f^med^, and CD24^med^CD49f^−/low^ populations. (XLSX 65 kb)

Additional file 4: **Figure S3.** Limiting dilution transplantation series in C57BL/6 and FVB mice. (A) Numbers of cells engrafted and ductal outgrowth data from limiting dilution transplantation series in C57BL6 mice from the indicated sorted populations. (B) Numbers of cells engrafted and ductal outgrowth data from limiting dilution transplantation series in FVB mice from the indicated sorted populations. (C) Summary of limiting dilution transplantation series from FVB mice. (D) Representative images of FVB-derived ductal outgrowths from the indicated populations. (E) Estimated frequency of ductal outgrowth forming cells in the indicated transplanted population from FVB mice. (PDF 487 kb)

Additional file 5: **Figure S4.** Thy-1^+^CD24^med^CD49f^high^ MRUs produce functional mammary epithelium. (A) Secondary transplant data from the indicated originally transplanted sorted population. (B) Tertiary transplant data from the indicated originally transplanted sorted population. (C) Hematoxylin and eosin and immunofluorescence staining of the indicated cytokeratin proteins in wild-type (WT) and serially transplanted Thy-1^+^CD24^med^CD49f^high^ epithelium. “Preg” denotes recipient mice that hosted donor ductal outgrowths that were mated and tissue analyzed at 11 days into pregnancy. (PDF 1889 kb)

## Abbreviations

MEC: Mammary epithelial cell
MRU: Mammary repopulating unit
ST-MRU: Short-term mammary repopulating unit
UMS: Ulnar mammary syndrome
WT: Wild-type
