# Supplementary figures and images for: Serially transplantable mammary epithelial cells express the Thy-1 antigen
Source: Breast Cancer Res. 2018 Oct 10;20:121. doi: 10.1186/s13058-018-1006-y (PMC6180607; doi:10.1186/s13058-018-1006-y)

Figure S1

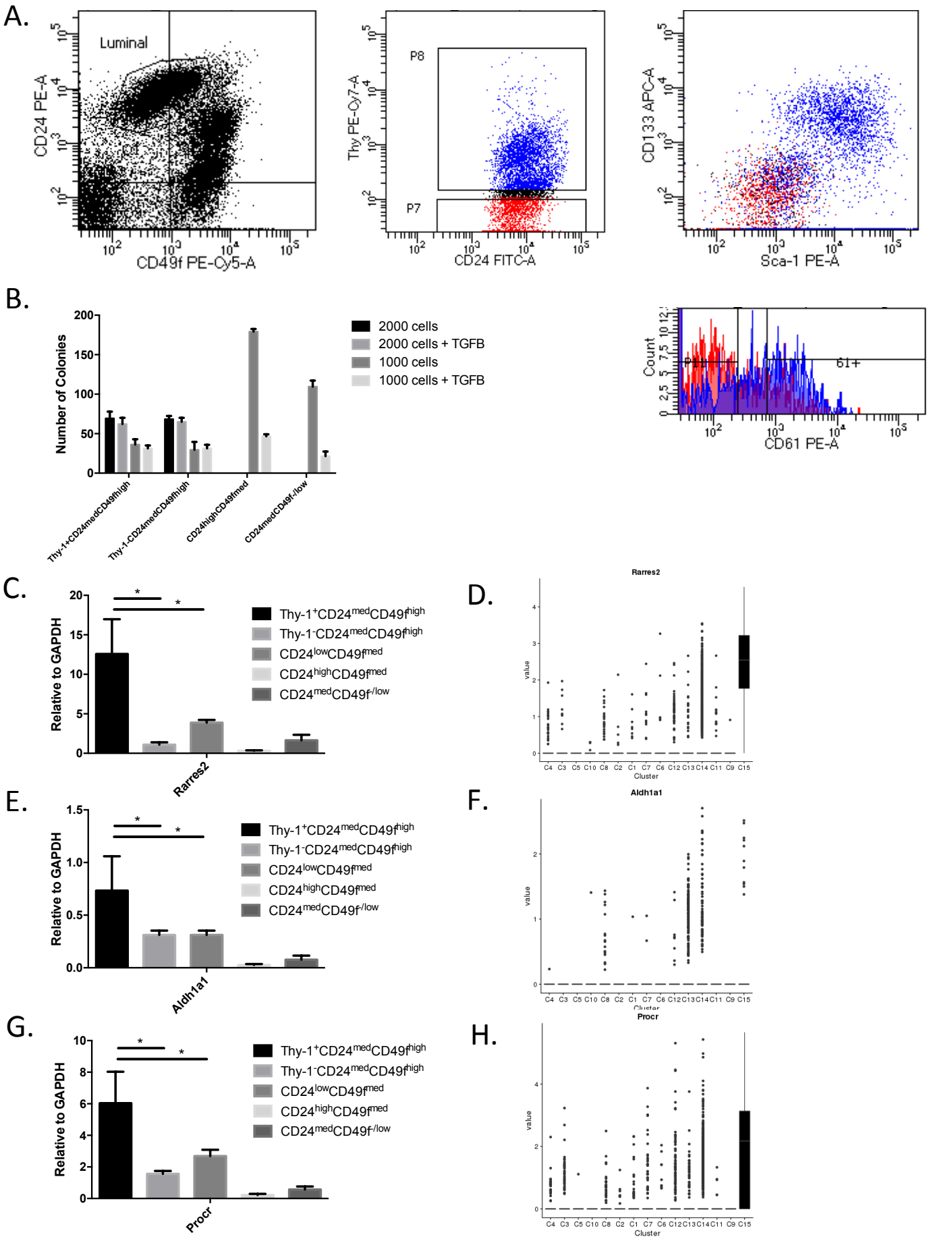

Supplement: Supplementary file 1 — Figure S1. Phenotypic and physiological analysis of sorted populations. (A) Flow cytometric analysis on luminal cells for Thy-1, CD133, Sca-1, and CD61 expression. (B) Exposure of cultured FACS-enriched mammary populations to TGFB1 ligand. The indicated sorted mammary populations were cultured in three-dimensional self-renewing conditions with and without the addition of 5 ng/ml human TGFB1 ligand. N = 3, ± STD. (C) Real-time PCR of sorted populations for Procr expression. N = 3, ± STD. *-p < 0.05 for an unpaired two-tailed t test. (D) Procr expression in mouse mammary epithelial from single-cell RNA-seq data using the web tool (http://marionilab.cruk.cam.ac.uk/mammaryGland/) from Bach et al. [24]. C15 cells are annotated as Procr-enriched cells. (E) Real-time PCR of sorted populations for Rarres2 expression. N = 3, ± STD. *p < 0.05 for an unpaired two-tailed t test. (F) Rarres2 expression in mouse mammary epithelial from single-cell RNA-seq data using the web tool (http://marionilab.cruk.cam.ac.uk/mammaryGland/) from Bach et al. [24]. C15 cells are annotated as Procr-enriched cells. (G) Real-time PCR of sorted populations for Aldh1a1 expression. N = 3, ± STD. *p < 0.05 for an unpaired two-tailed t test. (H) Aldh1a1 expression in mouse mammary epithelial from single-cell RNA-seq data using the web tool (http://marionilab.cruk.cam.ac.uk/mammaryGland/) from Bach et al. [24]. C15 cells are annotated as Procr-enriched cells. (PDF 293 kb) [file 13058_2018_1006_MOESM1_ESM.pdf]

Figure S2

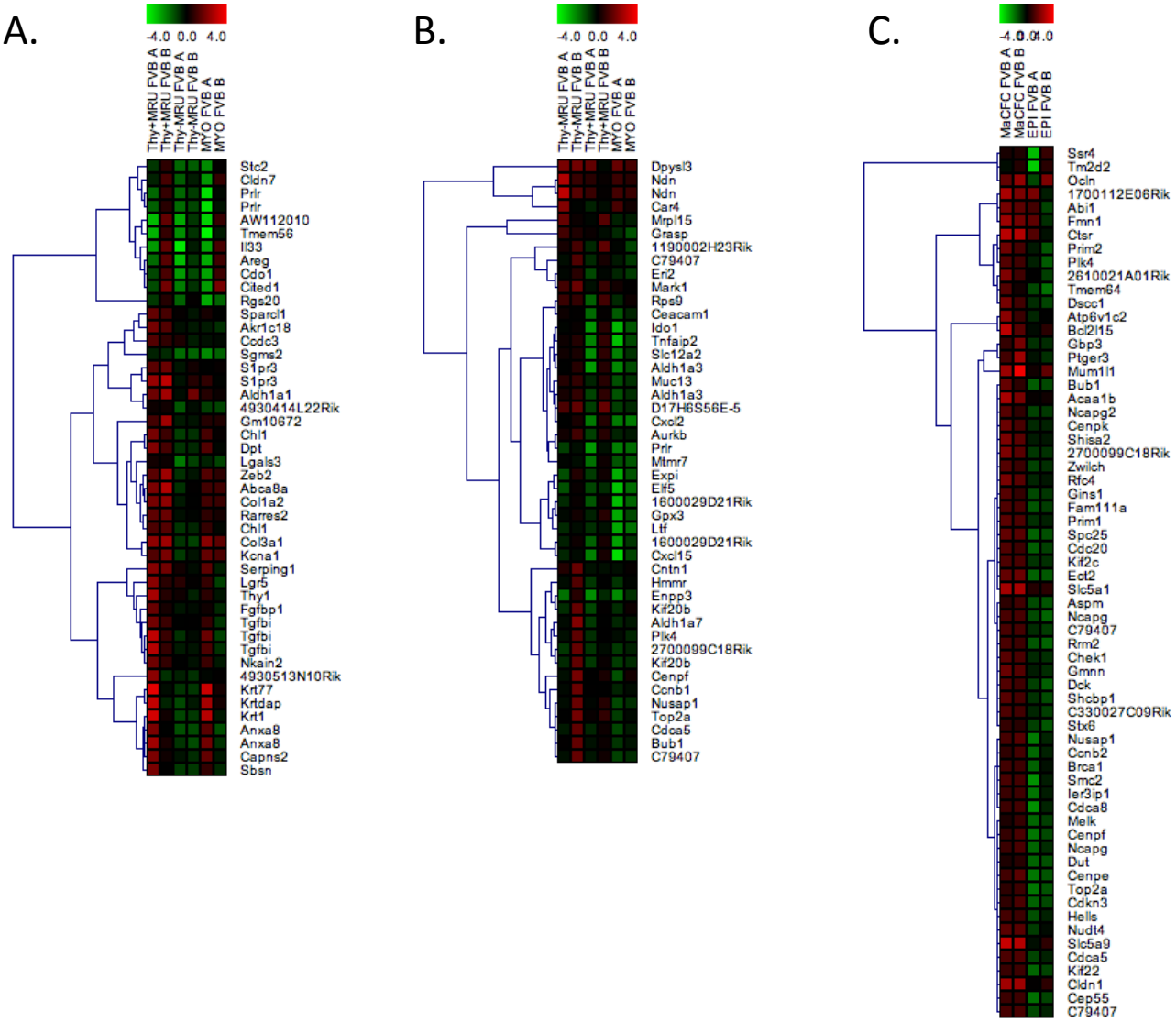

Supplement: Supplementary file 2 — Figure S2. Microarray analysis of phenotypically enriched populations isolated from virgin FVB female mice. (A) Differentially expressed probes comparing Thy-1+CD24medCD49fhigh to both Thy-1−CD24medCD49fhigh and CD24lowCD49fmed basal populations. (B) Differentially expressed probes comparing Thy-1−CD24medCD49fhigh to both Thy-1+CD24medCD49fhigh and CD24lowCD49fmed basal populations. (C) Differentially expressed probes comparing CD24highCD49fmed to CD24medCD49f−/low luminal cells. (PDF 186 kb) [file 13058_2018_1006_MOESM2_ESM.pdf]
