# Supplementary material for: Serially transplantable mammary epithelial cells express the Thy-1 antigen
Source: Breast Cancer Res. 2018 Oct 10;20:121. doi: 10.1186/s13058-018-1006-y (PMC6180607; doi:10.1186/s13058-018-1006-y)
Supplement: Supplementary file 4 — Figure S3. Limiting dilution transplantation series in C57BL/6 and FVB mice. (A) Numbers of cells engrafted and ductal outgrowth data from limiting dilution transplantation series in C57BL6 mice from the indicated sorted populations. (B) Numbers of cells engrafted and ductal outgrowth data from limiting dilution transplantation series in FVB mice from the indicated sorted populations. (C) Summary of limiting dilution transplantation series from FVB mice. (D) Representative images of FVB-derived ductal outgrowths from the indicated populations. (E) Estimated frequency of ductal outgrowth forming cells in the indicated transplanted population from FVB mice. (PDF 487 kb) [file 13058_2018_1006_MOESM4_ESM.pdf]

Figure S3

A.

C57BL6/J

Dose	Tested	Response	Group
200	11	5	Thy-1+CD24medCD49fhigh
100	44	25	Thy-1+CD24medCD49fhigh
50	31	13	Thy-1+CD24medCD49fhigh
30	15	12	Thy-1+CD24medCD49fhigh
5	29	7	Thy-1+CD24medCD49fhigh
200	5	0	Thy-1-CD24medCD49fhigh
100	30	2	Thy-1-CD24medCD49fhigh
50	24	1	Thy-1-CD24medCD49fhigh
30	9	0	Thy-1-CD24medCD49fhigh
5	30	2	Thy-1-CD24medCD49fhigh
200	11	5	CD24medCD49fhigh
100	13	4	CD24medCD49fhigh
5	14	2	CD24medCD49fhigh

B.

FVB

Dose	Tested	Response	Group
100	18	8	Thy-1+CD24medCD49fhigh
50	5	2	Thy-1+CD24medCD49fhigh
100	16	1	Thy-1-CD24medCD49fhigh
50	3	0	Thy-1-CD24medCD49fhigh

C.

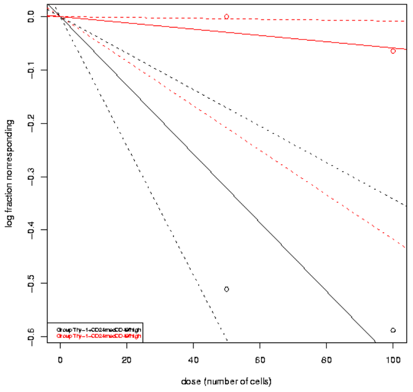

D.

Thy-1^{+}CD24^{med}CD49f^{high} Thy-1^{-}CD24^{med}CD49f^{high} CD24^{high}CD49f^{med}

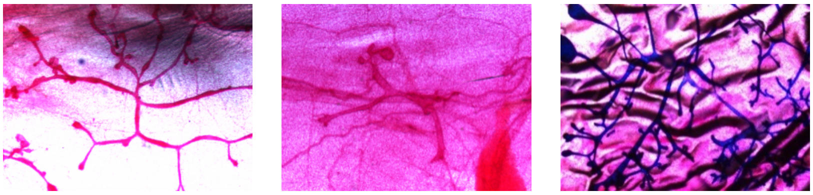

E.

Group	Lower	Estimate	Upper
Thy-1+CD24medCD49fhigh	293	156	83.1
Thy-1-CD24medCD49fhigh	12054	1700	240.1
