# Supplementary material for: Serially transplantable mammary epithelial cells express the Thy-1 antigen
Source: Breast Cancer Res. 2018 Oct 10;20:121. doi: 10.1186/s13058-018-1006-y (PMC6180607; doi:10.1186/s13058-018-1006-y)

Figure S4

A.

Original Transplanted Population	Engrafted/Transplanted	Efficiency
Lineage$^-$ 25K	10/11	91%
Thy-1^{med}CD24^{med}CD49f^{high} 600 cells	5/6	83%
Thy-1^{med}CD24^{med}CD49f^{high} 100 cells	6/10	60%
Thy-1^{med}CD24^{med}CD49f^{high} 30 cells	6/9	67%
Thy-1^{hi}CD24^{med}CD49f^{high} 100 cells	6/8	75%
Thy-1$^-$CD24^{med}CD49f^{high} cells	1/9	11%

B.

Original Transplanted Population	Engrafted/Transplanted	Efficiency
Lineage$^-$ 25K	14/14	100%
Thy-1^{med}CD24^{med}CD49f^{high} 100 cells	12/12	100%

C.

Thy-1$^+$CD24^{med}CD49f^{high}

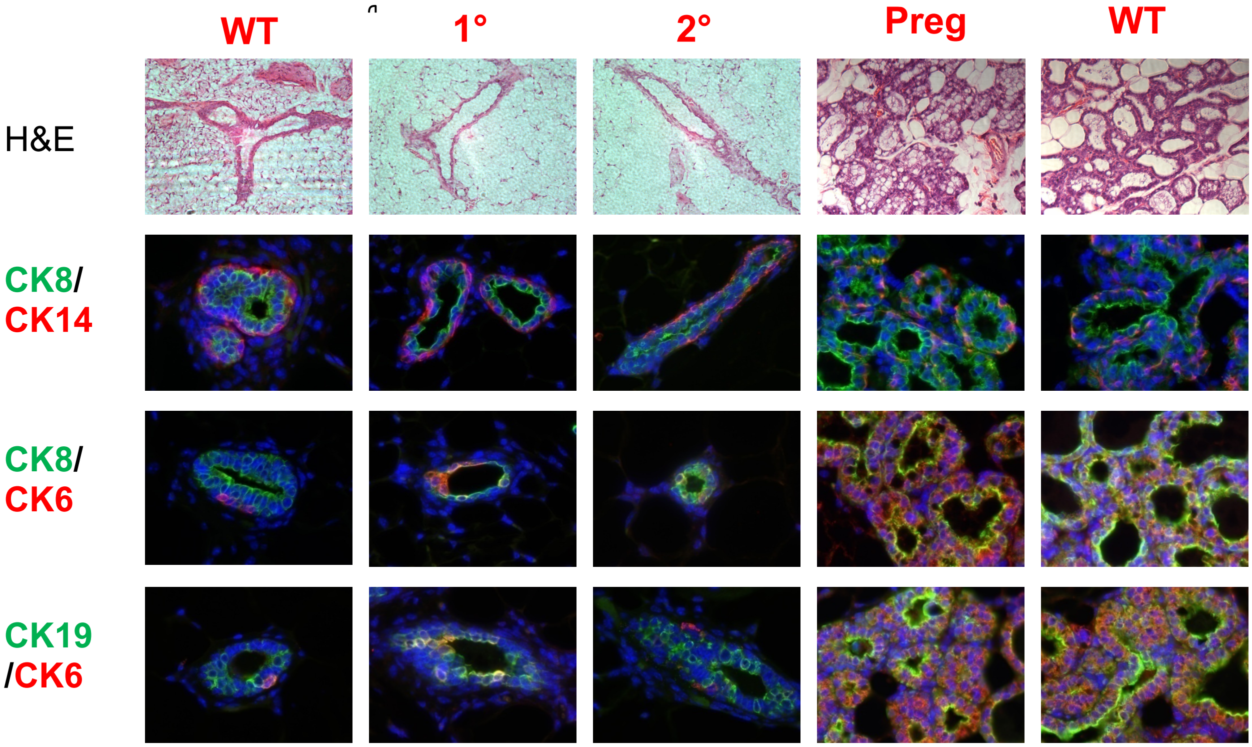

Supplement: Supplementary file 5 — Figure S4. Thy-1+CD24medCD49fhigh MRUs produce functional mammary epithelium. (A) Secondary transplant data from the indicated originally transplanted sorted population. (B) Tertiary transplant data from the indicated originally transplanted sorted population. (C) Hematoxylin and eosin and immunofluorescence staining of the indicated cytokeratin proteins in wild-type (WT) and serially transplanted Thy-1+CD24medCD49fhigh epithelium. “Preg” denotes recipient mice that hosted donor ductal outgrowths that were mated and tissue analyzed at 11 days into pregnancy. (PDF 1889 kb) [file 13058_2018_1006_MOESM5_ESM.pdf]
